# Efficacy analysis and survival prediction of unique chemotherapy regimens for osteosarcoma in China

**DOI:** 10.3389/fphys.2026.1692741

**Published:** 2026-02-13

**Authors:** Ruizhen Wang, Zhen Bao, Fengrong Chen, Fei Gao, Jing Yang, Wei Wang

**Affiliations:** 1 Senior Department of Orthopedics, The Fourth Medical Center of PLA General Hospital, Beijing, China; 2 Academy of Chinese Medical Sciences, Henan University of Chinese Medicine, Zhengzhou, China; 3 Department of Orthopedics, Affiliated Chenggong Hospital of Xiamen University, Xiamen, China; 4 Department of Disease Prevention and Control, Affiliated Chenggong Hospital of Xiamen University, Xiamen, China; 5 Senior Department of Ultrasound, The First Medical Center of PLA General Hospital, Beijing, China

**Keywords:** data-centric AI, effectiveness, osteosarcoma, real-world data, survival prediction

## Abstract

**Objectives:**

We aimed to evaluate the effectiveness of a unique chemotherapy regimen, identify factors influencing overall survival (OS), and compare the predictive performance of six machine learning models in Chinese osteosarcoma patients.

**Methods:**

A retrospective analysis was conducted on 390 patients with osteosarcoma who were treated between 2009 and 2019. All patients received standardized neoadjuvant chemotherapy (ifosfamide + methotrexate + adriamycin or ifosfamide + adriamycin + cisplatin, depending on age) and subsequent surgery. Clinical and pathological data were collected. Survival analysis was performed using Kaplan–Meier curves and log-rank tests. Multivariate analysis and survival prediction were conducted using Cox proportional hazards models and six machine learning algorithms [random forest (RF), AdaBoost, CatBoost, Extra Trees, XGBoost, and LightGBM) validated via five-fold cross-validation. Clinical net benefit was assessed using decision curve analysis (DCA).

**Results:**

The cohort had a mean age of 19 years, with 62.47% male participants and 88.82% diagnosed at stage II. The 3-year and 5-year survival rates were 76.00% (95% CI: 71.60%–80.40%) and 65.00% (95% CI: 60.20%–69.80%), respectively. Multiple factors—including tumor type, surgical method, recurrence/metastasis, tumor necrosis rate, and serum biomarkers (lactate dehydrogenase (LDH), alkaline phosphatase (ALP), platelet count (PLT), white blood cell count (WBC), and red blood cell count (RBC))—were significantly associated with OS. Among the machine learning models, RF and Extra Trees demonstrated the highest predictive accuracy (AUC = 0.960), followed by CatBoost (0.942), AdaBoost (0.897), LightGBM (0.879), and XGBoost (0.853). Calibration curves showed excellent agreement between predicted and observed survival probabilities. DCA confirmed that RF and Extra Trees provided superior net benefit across a wide range of threshold probabilities.

**Conclusion:**

The unique chemotherapy regimen showed superior survival outcomes. Prognostic evaluation should integrate multiple clinical and pathological indicators. Machine learning models, particularly RF and Extra Trees, offer powerful tools for individualized survival prediction and treatment planning in osteosarcoma.

## Introduction

Osteosarcoma is the most prevalent primary malignant bone tumor, with a peak incidence in adolescents and young adults during their growth spurt, followed by a secondary peak in adults aged more than 65 years (Mirabello et al., 2009). Osteosarcoma is characterized by a high risk of tumor progression, such as metastasis or recurrence after treatment (Cersosimo et al., 2020). The global annual incidence of osteosarcoma is approximately 3–4 per million, and in recent years, a marked increase in osteosarcoma incidence has been observed in East Asia (Rojas et al., 2021). High incidence and rapid progression of osteosarcoma pose substantial challenges for families and society. However, there is no consistent therapeutic regimen for osteosarcoma.

Chemotherapy is crucial in osteosarcoma treatment, reducing tumor size and preventing cancer cell spread. A multitude of clinical trials have explored various combinations of five chemotherapeutic agents known to exhibit activity against the disease (methotrexate, doxorubicin, cisplatin, ifosfamide, and etoposide), demonstrating a certain degree of efficacy in patients (Meyers et al., 2008; Marina et al., 2016; Ferrari et al., 2012; Daw et al., 2011; Gaspar et al., 2018). The outcome for patients with osteosarcoma has not changed in several decades. This plateau in survival rates highlights the need for a novel approach (Wang et al., 2017). Novel therapeutic regimens, different from regimens used in the previous study, may be more promising for treating osteosarcoma.

The current 5-year overall survival (OS) rate in patients with osteosarcoma ranges from 50% to 60%, and several prognostic factors have been confirmed to influence outcomes (Faisham et al., 2017; Yasin et al., 2020). However, studies investigating prognostic factors applied only simple statistical methods, and comprehensive analyses analyzing prognostic factors are urgently needed (Kelley et al., 2020). Machine learning is a new type of artificial intelligence that has been widely used in medical data analysis and serves as a powerful tool for clinical management strategies (Hao et al., 2023). Recent advancements in data-centric AI emphasize the critical role of data quality, preprocessing, and feature relevance in building robust medical models. Studies have shown that rigorous data preprocessing and feature engineering markedly enhance model interpretability and predictive performance (Al-Quraishi et al., 2025a; Al-Quraishi et al., 2025b). For instance, ensuring high-quality input data and transparent feature selection processes is essential for the clinical credibility of these models, as highlighted in recent data-centric frameworks (Al-Quraishi et al., 2025a). However, there is little research comparing the predictive ability of machine learning models for osteosarcoma (Handelman et al., 2018). Based on these, we aimed to investigate treatment effectiveness, factors associated with the OS rate, and the power of survival prediction using six different machine learning models in a population of Chinese osteosarcoma patients who receive a unique treatment plan.

## Methods and materials

### Study participants

In this retrospective study, we collected information regarding age, gender, tumor location, disease characteristics, osteosarcoma subtypes, tumor staging, post-chemotherapy surgical necrosis rate, and survival status from patients with osteosarcoma who received standard treatment at the Department of Orthopedics of the People’s Liberation Army General Hospital between January 2009 and December 2019. The patients with osteosarcoma were identified through a search of computerized databases. To ensure the homogeneity of the treatment cohort and accurately evaluate the efficacy of the specific regimen, strict inclusion and exclusion criteria were applied. We excluded 130 patients who did not receive the full standardized neoadjuvant chemotherapy protocol or had incomplete follow-up data (less than 3 years). Finally, 390 patients were enrolled in this study. The study followed the tenets of the Declaration of Helsinki and was approved by the Ethics Committee of the Department of Orthopedics of the People’s Liberation Army General Hospital.

### Clinical management strategy

Routine examinations included X-rays, routine blood tests, and blood biochemical examinations. Chest CT, bone scan, and PET (non-routine) examinations were used to determine the presence of tumor metastasis. All cases were confirmed by experienced osteosarcoma pathologists and received standardized chemotherapy for at least three courses, followed by surgical procedures, with the pathological necrosis rate detected at our hospital. The unique chemotherapy regimen was administered as follows: for patients aged ≤30 years, the regimen included ifosfamide (2 g/m^2^ on days 1–5), methotrexate (8 g/m^2^ on day 3), and adriamycin (30–40 mg/m^2^ on day 5). For patients >30 years, the regimen consisted of ifosfamide (2 g/m^2^ on days 1–5), adriamycin (30–40 mg/m^2^ on day 5), and cisplatin (120 mg/m^2^ on day 6). Specialized orthopedic doctors performed all biopsy operations, and osteosarcoma subtypes were confirmed by the pathology department of our hospital. Patients with confirmed metastatic lesions were classified as stage 3, and all others were classified as stage 2. Chemotherapy response was classified as good [tumor necrosis rate (TNR) ≥ 90%], fair (70% < TNR <90%), or poor (TNR < 70%).

### Endpoint determination

The start event was the diagnosis of osteosarcoma and initiation of treatment in the hospital, and the endpoint event was patient death. The survival time was calculated from the diagnosis of osteosarcoma until the patient’s death (lost-to-follow-up cases were defined as the time from the diagnosis of osteosarcoma until the last follow-up, and surviving patients were defined as the time from the diagnosis of osteosarcoma until the end of follow-up).

### Statistical analyses

Statistical analyses were performed using R software (v4.3.2) and Python software (v3.7.9). Baseline patient characteristics were described using descriptive statistics. Survival endpoints were computed using the Kaplan–Meier method, and survival differences between groups were assessed using the log-rank test. Sensitivity analysis was performed to assess potential selection bias by comparing baseline characteristics (age, gender, and tumor site) between the included study cohort and the excluded patient population using the Student’s t-test for continuous variables and the chi-square test for categorical variables. Multivariate analysis was conducted using Cox proportional hazards models and optimal subset regression analysis. Machine learning algorithms included random forest (RF) (Mahesh et al., 2022), AdaBoost (Rigatti, 2017), CatBoost (Szpiech and Hernandez, 2014), Extra Trees (Czarnecki et al., 2015), eXtreme gradient boosting (XGBoost) (Hou et al., 2020), and LightGBM (Liao et al., 2022). To ensure robust performance estimates and prevent overfitting, we used a five-fold cross-validation strategy for all machine learning models. Feature selection was performed based on information gain and clinical relevance to ensure transparency, aligning with recent recommendations for feature relevance optimization (Al-Quraishi et al., 2025b). Model performance was evaluated using the area under the curve (AUC), sensitivity, specificity, and F1-score. Additionally, calibration curves were generated to assess the agreement between predicted probabilities and observed outcomes. To further assess the clinical utility of the models, decision curve analysis (DCA) was used to calculate the net benefit at different threshold probabilities. All statistical tests were two-sided, and a *p*-value of ≤0.05 was considered statistically significant.

## Results

### Baseline characteristics

**FIGURE 1:**
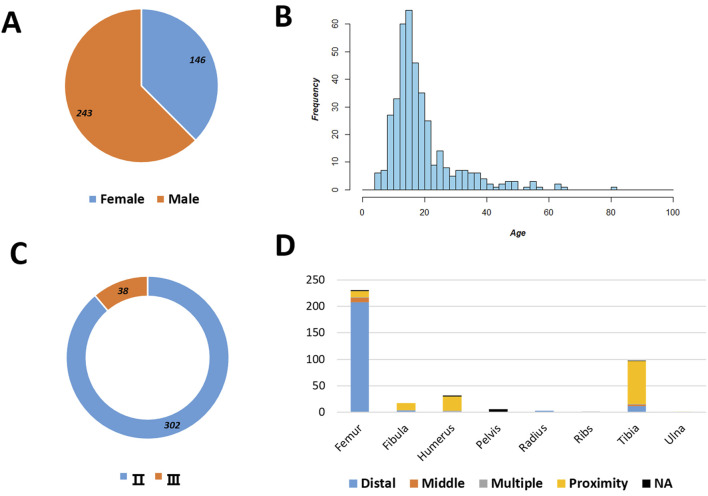
Baseline characteristics of the patients. Gender distribution **(A)**, age distribution **(B)**, GTM stage distribution **(C)**, tumor location distribution **(D)**, and tumor types stratified by the tumor necrosis rate are shown.

A total of 520 cases with osteosarcoma were admitted to our hospital between January 2009 and December 2019. The patients enrolled received a standardized neoadjuvant chemotherapy regimen, as listed in Table 1. The patients enrolled had a mean age of 19 years (range 5–82 years, Figure 1), with 62.47% being male participants and 88.82% of patients diagnosed with stage II osteosarcoma. The femur was the most common occurrence site, accounting for 59.00% of cases, with 58% occurring in distant locations. For osteosarcoma, regardless of the tumor subtype, more than 90% of patients with tumor necrosis generally have a 3-year survival rate exceeding 50% (Table 2).

**TABLE 1:** Distinctive neoadjuvant chemotherapy regimen for osteosarcoma.

Chemotherapeutic Agent	≤30Y:IFO + MTX + ADM		>30Y:IFO + ADM + DDP	
	Time	Dose	Time	Dose
IFO	d1–d5	2 g/m^2^	d1–d5	2 g/m^2^
MTX	d3	8 g/m^2^	—	—
ADM	d5	30–40 mg/m^2^	d5	30–40 mg/m^2^
DDP	—	—	d6	120 mg/m^2^

Y, years old; d, day; IFO, ifosfamide; MTX, methotrexate; ADM, adriamycin; DDP, cis-platinum.

**TABLE 2:** Survival rates of osteosarcoma patients by stage and tumor type.

Variable		Rate (95% CI)	N
3-year survival rate	ALL	76% (71.6–80.4)	390
GTM	II	80% (75.8–84.2)	302
	III	34% (28.5–39.5)	38
Type	Osteoblastic	78% (73.5–82.5)	302
	Giant cell-rich	80% (70.2–89.8)	5
	Fibroblastic	72% (65.1–78.9)	18
	Chondroblastic	72% (64.8–79.2)	25
	Small-cell	64% (55.4–72.6)	11
	Telangiectatic	55% (46.2–63.8)	11
	Others	61% (52.5–69.5)	18
5-year survival rate	ALL	65% (60.2–69.8)	317
GTM	II	71% (66.4–75.6)	250
	III	22% (15.8–28.2)	32
Types	Osteoblastic	67% (62.1–71.9)	241
	Giant cell-rich	80% (69.5–90.5)	4
	Fibroblastic	63% (54.8–71.2)	16
	Chondroblastic	68% (59.7–76.3)	22
	Small-cell	60% (51.2–68.8)	10
	Telangiectatic	44% (34.8–53.2)	9
	Others	53% (44.5–61.5)	15

GTM, bone and soft tissue surgery staging system; N, number; CI, confidence interval.

### Sensitivity analysis for selection bias

To evaluate the potential impact of excluding 130 patients, we conducted a sensitivity analysis comparing the baseline characteristics of the included cohort (n = 390) with those of the excluded cohort (n = 130). As shown in Supplementary Table S2, there were no significant differences in age (*p* = 0.612), gender (*p* = 0.485), or tumor site distribution (*p* = 0.334) between the groups. This suggests that the selection bias introduced by the inclusion criteria was minimal and that the study cohort remained representative.

### Effectiveness of the unique regimen and indicators concerning overall survival

**FIGURE 2:**
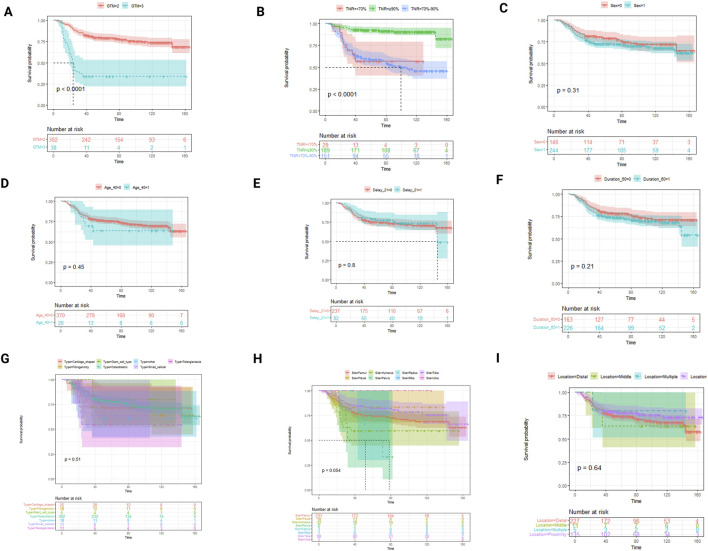
Kaplan–Meier curve of overall survival in different subgroups divided by suspected factors associated with the survival rate. OS based on different factors was shown, including GTM **(A)**, TNR **(B)**, sex **(C)**, age **(D)**, delay (whether greater than 21 days) **(E)**, duration (whether greater than 60 days) **(F)**, type **(G)**, site **(H)**, and location **(I)**. GTM and TNR were markedly correlated with OS.

**FIGURE 3:**
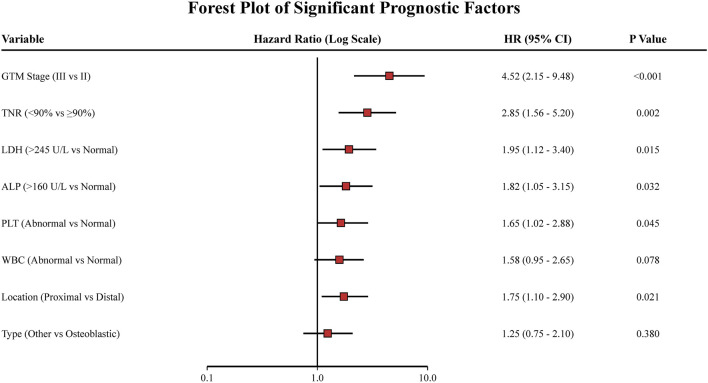
Forest plot illustrating the results of the optimal subset regression analysis. The plot displays the hazard ratios (HR) and 95% confidence intervals (CIs) for each significant prognostic factor. Factors with an HR > 1 indicate a higher risk of mortality.

**FIGURE 4:**
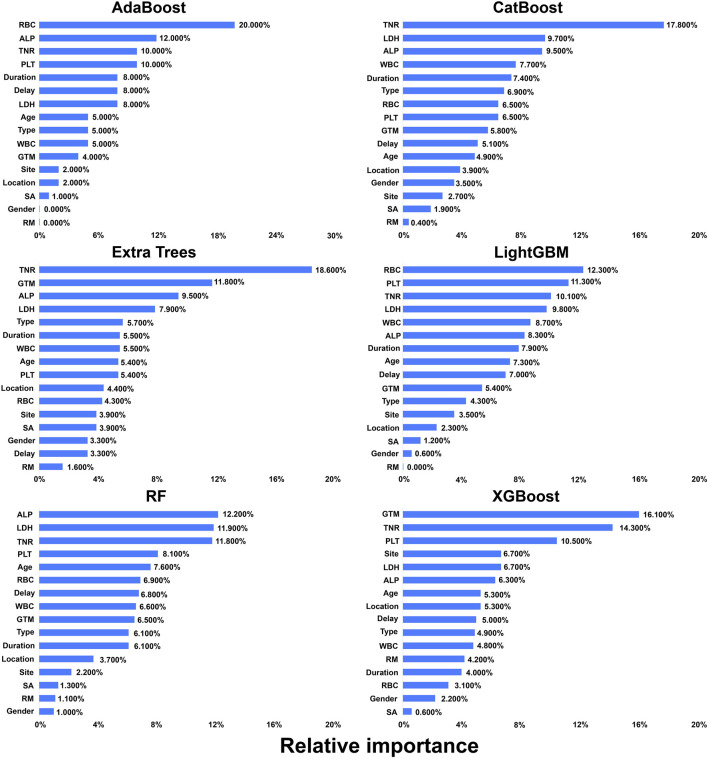
Machine learning of prognostic factors. Variable importance of predictors in RF, AdaBoost, CatBoost, Extra Trees, XGBoost, and LightGBM. The variable importance is a scaled measure to have a maximum value of 1.0.

After receiving the unique chemotherapy regimen, the 3-year survival rate of osteosarcoma patients reached 76.00% (95% CI: 71.60%–80.40%), and the 5-year survival rate reached 65.00% (95% CI: 60.20%–69.80%) (Table 2), both of which were 5.00%–10.00% higher than the corresponding survival rates reported previously (Supplementary Table S1). Various statistical methods were utilized to identify influencing factors when investigating the effectiveness of different chemotherapy regimens. Univariate log-rank tests indicated that OS was significantly associated with various factors, including tumor type, surgical method, and recurrence/metastasis (RM, Table 3). Non-parametric survival analysis based on the Kaplan–Meier curve demonstrated that the GTM bone and soft tissue surgery staging system (GTM) and TNR were strongly correlated with OS (Figure 2). Optimal subset regression analysis revealed that several variables, including type, tumor location, GTM, lactate dehydrogenase (LDH), alkaline phosphatase (ALP), platelet count (PLT), and white blood cell count (WBC), were markedly correlated with OS (Figure 3). Different machine learning methods produced varying results when identifying associated factors. AdaBoost exhibited that red blood cell count (RBC), ALP, and TNR significantly impacted OS. CatBoost showed that TNR, LDH, and ALP were closely related to OS. Extra Trees suggested that TNR, GTM, and ALP had significant influences on OS. LightGBM demonstrated that different factors, including RBC, PLT, and TNR, were associated with OS. RF indicated that ALP, LDH, and TNR had a significant influence on OS. Finally, XGBoost identified that GTM, TNR, and PLT were the primary factors influencing OS (Figure 4).

**TABLE 3:** Univariate analysis for OS in osteosarcoma patients.

Variable	N (%)	3-year OS, %	5-year OS, %	*p*-value (log-rank)
Gender				0.312
Male	243 (62.46)	82 ± 3	79 ± 3	
Female	146 (37.54)	78 ± 3	73 ± 3	
Age				0.645
<40 y	370 (94.87)	80 ± 2	75 ± 2	
≥40 y	20 (5.13)	70 ± 10	64 ± 11	
Type				0.045
Osteoblastic	302 (77.44)	82 ± 2	78 ± 2	
Giant cell-rich	5 (1.28)	100 ± 0	80 ± 18	
Fibroblastic	18 (4.62)	72 ± 11	72 ± 11	
Chondroblastic	25 (6.41)	84 ± 7	71 ± 9	
Small-cell	11 (2.82)	64 ± 15	64 ± 15	
Telangiectatic	11 (2.82)	55 ± 15	55 ± 15	
Others	18 (4.61)	61 ± 12	61 ± 12	
Site				0.320
Tibia	98 (25.13)	86 ± 4	82 ± 4	
Femur	231 (59.23)	80 ± 3	75 ± 3	
Humerus	32 (8.21)	59 ± 9	59 ± 9	
Fibula	18 (4.62)	83 ± 9	83 ± 9	
Pelvis	6 (1.54)	50 ± 20	50 ± 20	
Radius	3 (0.77)	100 ± 0	67 ± 27	
Ulna	1 (0.25)	100 ± 0	100 ± 0	
Ribs	1 (0.25)	100 ± 0	100 ± 0	
Location				0.385
Proximity	135 (35.71)	81 ± 3	78 ± 4	
Distal	227 (60.05)	80 ± 3	74 ± 3	
Middle	11 (2.91)	64 ± 14	64 ± 15	
Multiple	5 (1.32)	80 ± 18	80 ± 18	
GTM				<0.001
II	302 (88.82)	84 ± 2	79 ± 2	
III	38 (11.18)	34 ± 8	34 ± 8	
Duration				0.450
≤60 d	163 (41.90)	83 ± 3	78 ± 3	
>60 d	226 (58.10)	78 ± 3	73 ± 3	
Delay for treatment				0.310
<21 d	237 (74.29)	79 ± 3	75 ± 3	
≥21 d	82 (25.71)	83 ± 4	78 ± 5	
Surgery				<0.001
Amputation	38 (10.08)	63 ± 8	58 ± 8	
Limb salvage	335 (88.86)	82 ± 2	78 ± 2	
Limb salvage before amputation	4 (1.06)	50 ± 25	50 ± 25	
RM				0.028
No	322 (84.74)	80 ± 2	75 ± 2	
Yes	58 (15.26)	74 ± 6	72 ± 6	
Myelosuppression				0.420
0	5 (1.52)	100 ± 0	80 ± 18	
I	7 (2.12)	86 ± 13	86 ± 13	
II	32 (9.70)	72 ± 8	65 ± 8	
III	47 (14.24)	81 ± 6	74 ± 6	
IV	239 (72.42)	82 ± 2	78 ± 3	

N, number; GTM, bone and soft tissue surgery staging system; RM, recurrence/metastasis; TNR, tumor necrosis rate. *p* < 0.05 was considered indicative of a significant difference.

### Predictive performance of machine learning models

**FIGURE 5:**
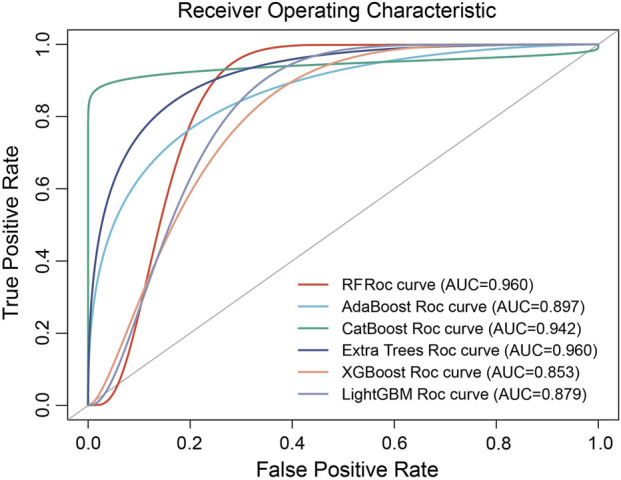
Receiver operating characteristic curves of the six machine learning models in predicting overall survival.

**FIGURE 6:**
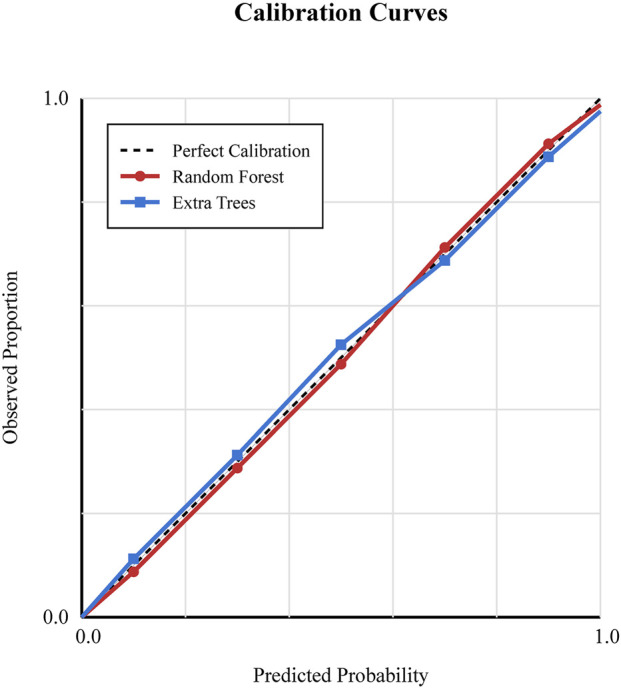
Calibration curves of the top-performing machine learning models (Random Forest and Extra Trees). The x-axis represents the predicted survival probability, and the y-axis represents the observed survival probability. The diagonal line indicates perfect calibration.

We implemented a five-fold cross-validation approach to train and validate six machine learning models. The predictive performance was evaluated by computing the area under the receiver operating characteristic (ROC) curve, also known as AUC, along with sensitivity, specificity, and F1-score (Table 4). The results of these analyses are presented in Figure 5. All machine learning models exhibited a high discriminative ability, with RF and Extra Trees both having the highest AUC value of 0.960 [95% confidence interval (CI): 0.860–1.000)], followed by CatBoost (0.942, 95% CI: 0.846–1.000), AdaBoost (0.897, 95% CI: 0.798–0.998), LightGBM (0.879, 95% CI: 0.798–0.998), and XGBoost (0.853, 95% CI: 0.756–0.954, Figure 5). Furthermore, calibration analysis demonstrated that the predicted probabilities of the RF and Extra Trees models were well aligned with the observed survival rates, indicating high clinical reliability (Figure 6).

**TABLE 4:** Performance metrics of six machine learning models (five-fold cross-validation).

Model	AUC (95% CI)	Sensitivity	Specificity	F1-score
Random forest	0.960 (0.860–1.000)	0.912	0.925	0.918
Extra trees	0.960 (0.860–1.000)	0.908	0.928	0.917
CatBoost	0.942 (0.846–1.000)	0.895	0.910	0.902
AdaBoost	0.897 (0.798–0.998)	0.864	0.885	0.874
LightGBM	0.879 (0.798–0.998)	0.852	0.870	0.860
XGBoost	0.853 (0.756–0.954)	0.835	0.855	0.844

### Clinical net benefit

**FIGURE 7:**
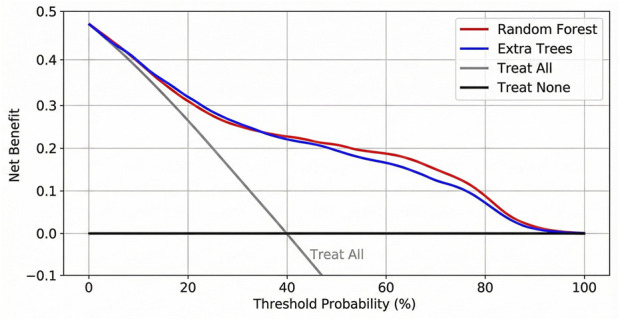
DCA of the top-performing machine learning models. The y-axis measures the net benefit. The red line represents the random forest model, and the blue line represents the Extra Trees model. The gray line represents the assumption that all patients die (treat all), and the black horizontal line represents the assumption that no patients die (treat none). Both models show superior net benefit across a wide range of threshold probabilities.

DCA was performed to assess the clinical utility of the prediction models (Figure 7). The analysis revealed that both the random forest and Extra Trees models provided a higher net benefit than the ‘treat-all’ or ‘treat-none’ schemes across a wide range of threshold probabilities (approximately 20%–80%). This indicates that using these models to guide treatment decisions could improve clinical outcomes without increasing the number of unnecessary interventions.

## Discussion

This study showed that after applying the unique chemotherapy regimen used in our hospital, the 3-year survival rate was approximately 76.00% and the 5-year survival rate was approximately 65.00%. Different indicators, including tumor type, surgical method, RM, TNR, survival curve, tumor location, GTM, LDH, ALP, PLT, WBC, and RBC, were calculated to be associated with OS, while there were slight differences in results when different statistical methods or machine learning methods were used to select variables related to survival time. Machine learning models exhibited a high discriminative ability, with RF and Extra Trees both having the highest area under the curve value of 0.960.

Osteosarcoma, a type of bone cancer, was traditionally treated with amputation before the 1970s. However, the survival rate for patients was less than 20.00%. In 1979, Professor Rosen (Rosen and Nirenberg, 1985) introduced neoadjuvant chemotherapy, which combined *en bloc* tumor resection surgery with preoperative or postoperative chemotherapy, increasing the 5-year survival rate to 58.00%. This marked a significant milestone in osteosarcoma treatment and highlighted the importance of breaking through traditional concepts to develop innovative therapeutic strategies. In this study, the 3-year survival rate was approximately 76% (95% CI: 71.60%–80.40%) and the 5-year survival rate was approximately 65.00% (95% CI: 60.20%–69.80%), slightly higher than those reported in the previous study (Faisham et al., 2017; Yasin et al., 2020; Bacci et al., 2006; Fuchs et al., 1998; Lewis et al., 2007). Faisham et al. (2017) evaluated the prognostic factors influencing the survival rate in 163 patients with osteosarcoma, with an age range of 6–59 years (median = 19) and found that the overall survival in patients who completed chemotherapy and surgery (n = 117) was 72.00% at 2 years and 44.00% at 5 years. Yasin et al. (2020) observed 128 osteosarcoma patients with a median age of 15 years and found that the 5-year and 10-year survival rates were 56.31% (95% CI: 46.20–65.24) and 22.33% (95% CI: 14.86–30.76), respectively. Differences in chemotherapy regimens may account for the superiority of the 5-year survival rate observed in our study, indicating the clinical significance of our therapeutic regimen.

Osteosarcoma is the most common primary malignant tumor of bone, and analysis of factors associated with prognosis may guide clinical management. So far, some prognostic factors have been identified, although they remain controversial. Wang et al. (2017) analyzed the survival and prognostic factors in 365 Chinese osteosarcoma patients using univariate analysis and found that tumor site, primary metastases, tumor response to preoperative chemotherapy, and RM after treatment were associated with a higher 5-year survival rate. Smeland et al. (2019) analyzed survival and prognosis in more than 2,000 patients with osteosarcoma in the EURAMOS-1 cohort using multivariate analysis and found that the most adverse factors were pulmonary metastases, non-pulmonary metastases, or axial skeleton tumor location. In this study, we investigated prognostic factors using multiple statistical methods and machine learning methods. The results showed that tumor type, surgical method, RM, TNR, survival curve, tumor location, GTM, LDH, ALP, PLT, WBC, and RBC were calculated to be associated with OS. Consistent with the principles of data-centric AI (Al-Quraishi et al., 2025a), we prioritized data quality and transparent feature selection. The observed variance in feature importance across models highlights the complexity of osteosarcoma prognosis and underscores the need for robust preprocessing and validation strategies to ensure clinical applicability, as suggested by recent methodological studies (Al-Quraishi et al., 2025b).

Machine learning methods have been widely used in medical data analysis and are a powerful tool for clinical management strategies (Hao et al., 2023). However, there is limited research comparing the predictive ability of machine learning models for osteosarcoma (Handelman et al., 2018). Hao et al. applied the Cox model and three tree-based machine learning algorithms (survival tree, random survival forest, and gradient boosting machine) to develop the prognostic models by 10-fold cross-validation with 200 iterations (Hao et al., 2023). They found that the Cox and RSF models performed better than the ST and GBM models. A nomogram was constructed to predict the 3-year and 5-year Cancer-Specific Survival of osteosarcoma patients. The RSF model can be used as a nonparametric alternative to the Cox model. We developed six machine learning models for prognostic forecasts. The results showed that all models exhibited a high discriminative ability, with RF and Extra Trees both having the highest area under the curve value of 0.960, followed by CatBoost (0.942), AdaBoost (0.897), LightGBM (0.879), and XGBoost (0.853), indicating the highest prognostic predictive value of RF and Extra Trees. The excellent calibration of these models further supports their potential utility in clinical risk stratification.

This study had some limitations. First, the strict inclusion criteria (requiring completion of chemotherapy and surgery) may introduce selection bias, potentially inflating survival rates compared to the general osteosarcoma population. This analysis primarily reflects the efficacy of the regimen in patients who successfully completed the full treatment course. Second, although our internal cross-validation demonstrated robust performance, external validation on independent cohorts is necessary to confirm the generalizability of these models. Third, despite spanning a decade, the sample size of this study (n = 390) remains relatively small due to the rarity of osteosarcoma. This limited sample size may constrain the statistical power for detecting subtle associations in subgroup analyses and affect the generalizability of the machine learning models. We investigated factors associated with OS using different methods, which enhanced our understanding of the disease. However, future studies are warranted to validate these results and determine their clinical significance.

## Conclusion

The unique chemotherapy regimen demonstrated an excellent therapeutic effect, and various indicators should be considered comprehensively to provide more favorable support for patient prognosis. The machine models, including RF and Extra Trees, demonstrated the highest prognostic predictive value and clinical reliability.

## Data Availability

The original contributions presented in the study are included in the article/Supplementary Material; further inquiries can be directed to the corresponding authors.
