# Supplementary material for: Efficacy analysis and survival prediction of unique chemotherapy regimens for osteosarcoma in China
Source: Front Physiol. 2026 Feb 13;17:1692741. doi: 10.3389/fphys.2026.1692741 (PMC12945764; doi:10.3389/fphys.2026.1692741)
Supplement: Supplementary file 1 [file Table1.docx]

**Supplementary Table 1. The TNR in different type of** **Osteosarcoma.**

| Type TNR | <70% | ≥90% | 70%-90% | All |
| --- | --- | --- | --- | --- |
| Osteoblastic | 18 | 159 | 111 | 288 |
| Cartilage_shaped | 2 | 7 | 15 | 24 |
| Fibrogenicity | 1 | 7 | 10 | 18 |
| Giant_cell_type | 0 | 2 | 3 | 5 |
| Small_cellular | 2 | 4 | 4 | 10 |
| Telangiectasia | 4 | 3 | 4 | 11 |
| other | 2 | 7 | 4 | 13 |
| All | 29 | 189 | 151 | 369 |
