# Supplementary material for: Efficacy analysis and survival prediction of unique chemotherapy regimens for osteosarcoma in China
Source: Front Physiol. 2026 Feb 13;17:1692741. doi: 10.3389/fphys.2026.1692741 (PMC12945764; doi:10.3389/fphys.2026.1692741)
Supplement: Supplementary file 2 [file Table2.docx]

**Supplementary Table 2: Sensitivity Analysis Comparing Baseline Characteristics of Included and Excluded Patients.**

| **Characteristic** | **Included Cohort (n=390)** | **Excluded Cohort (n=130)** | **P Value** |
| --- | --- | --- | --- |
| **Age (years), Mean ± SD** | 19.2 ± 12.5 | 19.8 ± 13.1 | 0.612 |
| **Gender, n (%)** |  |  | 0.485 |
| Male | 243 (62.3%) | 76 (58.5%) |  |
| Female | 147 (37.7%) | 54 (41.5%) |  |
| **Tumor Site, n (%)** |  |  | 0.334 |
| Femur | 231 (59.2%) | 72 (55.4%) |  |
| Tibia | 98 (25.1%) | 36 (27.7%) |  |
| Other | 61 (15.6%) | 22 (16.9%) |  |
